# Epidemiological and Clinical Characteristics of Adult and Pediatric Patients with Chronic Spontaneous Urticaria

**DOI:** 10.3390/jcm12237482

**Published:** 2023-12-03

**Authors:** Aviv Barzilai, Alona Baum, Moshe Ben-Shoshan, Ido Tzanani, Reman Hakroush, Dan Coster, Michal Solomon, Shoshana Greenberger

**Affiliations:** 1 Department of Dermatology, Sheba Medical Center, Ramat-Gan 52621, Israelhakreman@gmail.com (R.H.); solomomdr1@gmail.com (M.S.); 2 Institute of Pathology, Sheba Medical Center, Ramat Gan 52621, Israel; 3 Faculty of Medicine, Tel Aviv University, Tel Aviv 69978, Israel; 4 Division of Pediatric Allergy and Clinical Immunology, Department of Pediatrics, McGill University Health Center, Montreal, QC H4A 3J1, Canada

**Keywords:** chronic spontaneous urticaria, autoimmune co-morbidities, atopic diseases, second-generation antihistamine, Omalizumab

## Abstract

Chronic spontaneous urticaria (CSU) is when lesions occur for ≥6 weeks. However, its underlying mechanism remains unclear. CSU prevalence is similar in adult and pediatric patients; nevertheless, few data are available on CSU characteristics in pediatric patients. We aimed to describe the epidemiology, clinical features, and treatment approach of CSU in pediatrics and adults. In this cross-sectional study, 193 patients with CSU were treated at the Sheba Medical Center, Israel, in 2009–2022. The information collected includes age at diagnosis, reported triggers, atopic co-morbidities, autoimmune co-morbidities, treatments and their response, family background, laboratory tests, and follow-up duration. The study group was divided into pediatrics (aged ≤ 18) and adults. Metabolic syndrome was most prevalent in adults as against atopy in pediatrics. Autoimmune co-morbidities were observed in 34.7% and 34.8% of adults and pediatrics, respectively. Inflammatory bowel disease and thyroid disease were the most common in pediatrics and adults, respectively. Systemic treatments other than antihistamines were administered more frequently in adults. Adults with autoimmune disease required second-line treatment with immunomodulators compared to those without it. Co-morbidities were more common in adults than in pediatrics. Patients with autoimmune co-morbidities may be more challenging to manage; thus, escalation to biologics should be considered soon.

## 1. Introduction

Acute urticaria is common and affects approximately 20% of the population [1]. Urticaria lasting ≥6 weeks without an obvious trigger is defined as chronic spontaneous urticaria (CSU). The prevalence of CSU is reported to be 0.5–1% in the general population. Most patients with CSU are aged 20–40 [2]. Individual lesions fade within 24 h as new lesions continue to appear. The pruritus of CSU is associated with depression and anxiety and affects patients’ quality of life by interfering with sleep and normal daily activities. [3,4].

CSU is associated with various co-morbidities. Studies have also reported its association with the increased prevalence of atopic and autoimmune diseases [5]. In a Scandinavian study of 158 patients with CSU, 42% had atopic diseases, including atopic dermatitis, asthma, and conjunctivitis. Additionally, thyroid disease, hypertension, and obesity were reported in 5%, 7%, and 7% of the patients, respectively [6].

In a study in Taiwan, the same pattern of co-morbidities was observed, with an increased prevalence of drug allergies, rheumatic and inflammatory diseases, cancer, and psychiatric disorders compared with the standard population [7]. A recent systematic review revealed that thyroid diseases and vitiligo were the most prevalent autoimmune co-morbidities (>2% in patients with CSU), whereas anti-thyroid and anti-nuclear antibodies were predominant (>15% in patients with CSU) [8].

However, the pathogenesis of CSU is not fully understood. Recent studies have suggested that CSU is an autoimmune disease targeting mast cells [7]. In some patients, autoreactive immunoglobulin G that crosslinked immunoglobulin E (IgE) or IgE receptor on the mast cell surface causes degranulation. However, the other patients displayed no disease etiology. Evidence suggests that IgE autoantibodies, particularly IgE-targeting interleukin 24, may contribute to CSU pathogenesis [9].

The clinical diagnosis of CSU is mostly based on a thorough history and physical examination. Most CSUs are autoimmune-related and not due to an external trigger; therefore, limited laboratory tests are required for diagnosis. Nonetheless, recent guidelines recommend complete blood count (CBC), C-reactive protein (CRP), anti-thyroid peroxidase, and total IgE. In rare cases, when the patient’s history suggests parasitic infection, blood counts for IgE levels, eosinophilia, and stool cultures are beneficial. However, when urticarial vasculitis is suspected, assessment of collagen disease, including anti-nuclear antibodies and complement levels, together with skin biopsy, is indicated. For virus-associated CSU, Hepatitis B and C are crucial. In some cases, Helicobacter pylori infection is a possible etiology for CSU [1].

CSU has no cure; thus, treatment aims to control the disease. In addition to preventing known triggers that aggravate inducible CSU (sweat, heat, pressure, and sun), the first-line treatment for all patients with CSU is second-generation antihistamines (sgAHs), with doses that can be increased up to four times the standard. Treatment with Omalizumab and monoclonal anti-IgE is recommended for severe cases that are not well controlled by high-dose antihistamines [1]. Steroids are administered only for a brief period in CSU exacerbation. Cyclosporine and Methotrexate are alternative treatments [10].

The treatment guidelines for pediatric patients were extrapolated based on adult data [11,12]. Generally, a stepwise treatment algorithm similar to that for adults exists. However, Omalizumab is only approved for pediatric patients aged ≥ 12 because its efficacy and safety have not been widely assessed in those aged < 12 years. Increased antihistamine doses induce remission in up to 90% of pediatric patients. A recent review from Montreal, Canada, described treatment options for pediatric patients with CSU and highlighted the gaps in those < 12 years. The study demonstrated that Omalizumab is safe and effective for refractory CSU in pediatric patients, even in those aged < 12. In addition, Cyclosporine should only be considered in pediatric patients after Omalizumab treatment fails [13]. Of note, unlike most data on Omalizumab for treatment of CSU, Cyclosporine can induce long remissions after treatment cessation [14].

The prevalence of CSU in pediatric patients is 0.5–1.5%, similar to that reported in adults [15]. However, pediatric patients with CSU have a higher prevalence of atopic background (58%) than adults, and the prevalence of associated autoimmune diseases is similar in both age groups [16]. Data on the associated autoimmune diseases in pediatric patients with CSU are scarce. A recent study revealed that thyroid diseases and vitiligo were the most common autoimmune diseases in 1200 patients with CSU and associated autoimmune co-morbidities, with six patients (0.5%) aged <20 [17].

This study aimed to describe the epidemiology, underlying conditions, autoimmunity, clinical features, and treatment modalities of CSU in pediatric and adult patients.

## 2. Materials and Methods

This cross-sectional study included Israeli patients of all ages who were hospitalized in the dermatology department or treated at the dermatology outpatient clinic of the Sheba Medical Center in 2009–2022. The participants were clinically diagnosed with chronic urticaria with no obvious triggers and divided into two groups based on age. The adult and pediatric groups had patients > 18 years and ≤18 years, respectively. The inclusion criteria for the study were:

1. Confirmed clinical diagnosis of severe CSU (lesions lasting > 6 weeks). as evaluated by an expert dermatologist.
2. Patients with CSU who were hospitalized or examined as outpatients at Sheba Medical Center, with a follow-up of ≥3 months.
3. Patients with CSU with no obvious trigger.
4. Patients with CSU with complete background data on co-morbidities, including autoimmunity.

The following patients with CSU were excluded from the study: those with a follow-up period of <3 months, those whose CSU diagnosis was unclear, whose trigger, including active infection, might cause CSU, or patients with missing data regarding autoimmunity.

### Statistical Analysis

Data were analyzed using R software version 4.2.1. Continuous variables were measured as mean and standard deviations, whereas categorical variables were presented as proportions and percentages. A comparative analysis was performed between adults and pediatric groups using the Mann–Whitney–Wilcoxon test and Pearson’s χ2 test for continuous and categorical variables, respectively. A similar analysis was performed between participants with and without autoimmune co-morbidities using the same hypothesis tests. All statistical tests were two-tailed, and *p*-values of <0.05 were considered significant.

## 3. Results

Data were collected from 205 patients diagnosed with CSU, 193 of whom were included in this study. Twelve patients were excluded owing to documented chronic urticaria presentation of < 6 weeks or urticaria due to known triggers. In total, 170 patients were adults (116 females and 54 males), whereas 23 were pediatric (17 females and 6 males). The mean age at diagnosis for the adult and pediatric groups was 45.7 (±16.9) and 12.9 (±5.03) years, respectively. Overall mean follow-up was 19.1 months (±33.2). Co-morbidities other than autoimmune or atopic were observed in 48.8% of the adult group and 8.7% of the pediatric group (*p* = 0.001). Atopic diseases, such as asthma, allergic rhinitis, and conjunctivitis, were observed in 34 (20%) and 7 (30.4%) adult and pediatric patients, respectively. A family history of atopic co-morbidities was reported in 30 (17.6%) and 4 (17.4%) patients in the adult and pediatric groups, respectively. Eight adult (4.7%) patients used aspirin.

Angioedema was observed in addition to urticaria in 52.3% of all patients: 93 (54.7%) adult patients and 8 (34.8%) pediatric patients. In the pediatric group, chronic inducible urticaria (CindU), together with CSU, was observed in 7 (30.4%) patients (physical component). This was much greater than that in the adult group, which had only 16 patients (9.4%) with a physical component. In contrast, CindU with a hormonal component occurred only in adults. Table 1 summarizes other demographic and clinical characteristics and background diseases.

Overall, 67 patients (34.7%) had autoimmune co-morbidities: 59 (34.7%) were adults, and 8 (34.8%) were children (*p* = 1). We observed a significant difference between both groups for autoimmune liver disease and inflammatory bowel disease (IBD), which were prevalent in the pediatric group. Table 2 presents other autoimmune disease details and their markers.

Generally, 44% of all patients had a high CRP level (with no evidence of active infection), with no significant difference between the adult and pediatric groups (47.6% and 17.4%, respectively, *p* = 0.575). Other blood test results (CBC, IgE, TSH, and stool parasites) were within laboratory reference ranges. The laboratory findings revealed significant differences between both groups for thrombocytosis. Additionally, high TSH levels were observed in the pediatric group (8.7%, *p* = 0.05). Table 3 summarizes the complete data.

Steroids and antihistamines were the main treatment options. sgAH (desloratadine and fexofenadine) were more frequently administered than first-generation antihistamines (fgAH) (chlorphenamine and promethazine) in both groups. Furthermore, fgAH was frequently administered in the adult group (48.8% vs. 8.7%, *p* = 0.001). Most patients were treated twice or four times daily (38.8% or 32.9%, respectively). Oral and intravenous (IV) routes were used more often in the adult group (Oral: 70% vs. 39.1%, *p* = 0.007 and IV: 35.3% vs. 0%, *p* = 0.001). Systemic immunosuppressive treatments, including Methotrexate, Azathioprine, and Cyclosporine, were administered only to the adult group. Biological treatment (Omalizumab) was administered to both groups, with no significant difference (24.7% vs. 17.4% in the adult and pediatric groups, respectively, *p* = 0.6). Table 4 provides the treatment details.

Adults with an autoimmune background required second-line systemic therapies more often than those without it (71.2% vs. 53.2%, *p* = 0.03).

## 4. Discussion

In this study, we described the epidemiology, background diseases, clinical features, and treatment modalities of CSU in pediatric and adult patients. We included 193 patients with CSU in the study; 170 were adults, and 23 were pediatrics. Females accounted for approximately 68% of the adult group. These findings align with the literature, which states that CSU is twice as common in females [18,19,20]. Data regarding the incidence ratio of CSU in pediatric patients are scarce; nevertheless, sex predilection data report an equal incidence between the two sexes [21]. We observed a female predilection (73.9%) in the pediatric group, which could be explained by their higher mean age of diagnosis (15 years), which was younger in other studies [12,22]. The mean age at diagnosis in the adult group was 45.7 years, consistent with the literature [19,20,22]. However, the mean age (12.9 years) at diagnosis in the pediatric group was higher than that reported in previous studies [12].

Metabolic syndrome was the most common underlying condition in adults (35.9%). A similar prevalence rate was documented in a Republic of Korea study [22,23]. This outcome is higher than the prevalence reported worldwide (12.5–31.4%) and in Israel (10.6–20.2%) according to the definition considered [24,25].

An atopic background is associated with CSU and shares pathogenic pathways. For example, high IgE levels are observed in both diseases. Here, 34 (20%) patients in the adult group had an atopic background. In another study from Israel, the atopic background prevalence in patients with CSU corresponds with our findings for asthma (10.8% vs. 8.8%); however, a higher incidence of atopic dermatitis (9.8% vs. 5.3%) and allergic rhinitis (19.9% vs. 8.8%) was recorded. This difference can be explained by the small size of our research group, and further larger-scale studies will provide more information [26]. Furthermore, we observed atopy in seven pediatric patients (30.4%), consistent with earlier studies [21]. The higher prevalence of atopy in the pediatric group than in the adult group is explained by the higher incidence of the atopic march in younger age groups.

### 4.1. Clinical Presentation

Angioedema is a common symptom that often manifests with urticaria. It involves swelling of the sub-dermis of soft tissues, such as the eyelids, lips, tongue, and throat. Angioedema prevalence is approximately 33–67% in adults with CSU [19,20,27,28]., consistent with our outcome (54.7%) in CSU-associated angioedema. In pediatric patients, the prevalence of angioedema varies and is approximately 50% [12]. Our study revealed that the prevalence was 34.8%—lower than that reported previously—probably because of the small sample size of the pediatric group.

CSU can co-exist with CindU, most commonly in dermographism. Less common triggers include delayed pressure induced urticaria, cold, heat and exercise [1]. Literature reports on the prevalence of CSU with CindU vary (10–50%) [28,29]. We observed that the pediatric group had a significantly higher incidence of CindU than the adult group (30.4% and 9.4%, respectively; *p* = 0.01). Common triggers of CindU include rubbing and scratching. Children have a higher incidence of atopy; thus, we assumed dermographism was secondary to atopic pruritus. Moreover, only patients in the adult group had CindU levels with hormonal components associated with CSU. The role of sex hormones in CSU is unclear, and the hypothesis of their role in the pathogenesis is due to women suffering more frequently from CSU. Autoimmunity is associated with CSU and is induced by sex hormones [30].

### 4.2. Autoimmune Co-Morbidities

A strong association exists between CSU and other autoimmune diseases (AID), such as thyroid diseases, rheumatoid arthritis, systemic lupus erythematosus, celiac disease, and type 1 diabetes. The incidence of autoimmune thyroid diseases in patients with CSU varies in different series (7–57%) [31,32,33]. In a large-scale study of 2523 CU patients from Greece, autoimmune thyropathy was reported in 7.3% [20]. Patients with CSU and thyroid disease have a more complicated and prolonged disease course. Moreover, they have a higher risk of developing angioedema, estimated to be 16 times more common in patients with CSU without autoimmune thyroid diseases [31]. Furthermore, autoimmune thyroid diseases were the most prevalent AID in adults, accounting for 9.9% of patients (10% and 8.6% in the adult and pediatric groups, respectively). The association between CSU pathogenesis and thyroid autoimmunity is unclear. It is hypothesized that the association is linked to a genetic predilection to autoimmunity and some mechanisms that activate a domain in the complement protein C4, which reacts in the pathway of both diseases. Furthermore, patients with CSU who are clinically euthyroid have increased levels of anti-thyroid peroxidase antibodies (TPO Ab) [32]. Najafipour et al. reported that TPO was observed in 25–30% of patients with CSU, similar to our study findings [33].

Other AIDs have been reported in adults with CSU. In our study group, the incidence of AIDs and/or autoantibodies was 34.7% (59 adults), of which 22 (12.9%) had an autoimmune disease (Table 2). These outcomes are consistent with those reported in the literature [34].

Studies on the prevalence of AID among pediatric patients with CSU are scarce. Le et al. demonstrated that 10 of 191 Canadian pediatric patients with CSU had AID (hypothyroidism, type 1 diabetes, juvenile rheumatoid arthritis, and lupus) [35]. Our pediatric group comprised six (26%) patients, including one with two AIDs (IBD and autoimmune liver disease), two with IBD, and two with autoimmune thyroid illness. Autoimmune liver disease and IBD were more prevalent in the pediatric group than in the adult group. Two children not diagnosed with AIDs had autoimmune markers—one had positive anti-nuclear antibodies, and the other had positive perinuclear ANCA and anti-TPO Antibodies. The prevalence of AIDs among children is generally low, and its incidence increases with age. In a recent large study from Canada, AID prevalence was <5% among children with CSU [35]. Here, the incidence was higher (26%, *p* = 0.17), which could be explained by the small size of the pediatric group, and the children with autoimmunity were aged 10–18 years. To the best of our knowledge, this is the first study describing an association between autoimmune liver diseases and CSU in children.

### 4.3. Blood Tests Results

Different types of immunological events are associated with CSU, including mast cell activation and degranulation, increased coagulation activity, and an acute-phase response. CRP is supported by strong evidence that distinguishes patients with CSU from the general population [36]. CRP levels were elevated in both study groups (47.6% and 17.4% in adults and pediatrics, respectively, *p* = 0.57).

Thrombocytosis was also significantly elevated in the pediatric group (8.7%, *p* = 0.009). This finding corroborates elevated levels of acute-phase reactants in patients with CSU.

### 4.4. Treatment Protocol

Non-sedating sgAH up to four times daily is the mainstay treatment. In the acute phase of CSU, steroid therapies were more common among the adults using intravenous (*p* = 0.001) and oral (*p* = 0.007) administration than in pediatrics.

Based on a recent review from Canada, the mainstay treatment for pediatric patients with CSU is non-sedating sgAH. The available data regarding Omalizumab as a treatment for pediatric patients with CSU are promising. This treatment is currently being used and analyzed for further investigations in pediatrics [13].

sgAH was administered as first-line treatment in both groups in our study. Most patients were treated twice or four times daily (38.8% and 32.9%, respectively). Notably, treatment outcomes were better in our pediatric group than in the adult group, and the majority (65.2%) were remitted on antihistamines twice daily. Only four children (17.4%) required second-line treatment with Omalizumab, while 50% of the adult group required antihistamines four times a day, and 24.7% required second-line treatment with Omalizumab. Generally, systemic treatments other than antihistamines or steroids were more significantly used in the adult group (*p* = 0.03). Further statistical analysis of the adult group revealed that patients with autoimmune co-morbidities may be more challenging to manage, requiring continuous systemic treatment than adults without autoimmune co-morbidities (71.2% vs. 53%, respectively; *p* = 0.03). This supports the known data from the literature on patients with CSU with an autoimmune background [28]. However, the mechanism by which treating autoimmune diseases is more challenging remains unclear. We hypothesize that autoimmune diathesis can cause an unexpected immune response that requires treatment line escalation.

This study had several limitations. First, it included relatively few pediatric patients with CSU. Second, data were retrieved from a tertiary center record that might reflect more severe cases of CSU because milder cases might have been treated only in outpatient clinics, affecting the generalizability of the study. Furthermore, no standard CSU scores were documented.

## 5. Conclusions

To our knowledge, our study is the largest to compare pediatric and adult patients with CSU assessed in Israel. We conclude that children with CSU have a higher incidence of atopy and autoimmune diseases. Adults with CSU have higher incidences of metabolic syndrome and autoantibodies. Patients with CSU and autoimmune co-morbidities present a more complex disease, necessitating earlier treatment escalation for effective management. Larger randomized controlled studies should be conducted in adults and children with CSU.

## Figures and Tables

**Table 1:** Epidemiological characteristics, medical history, and clinical presentation.

Parameter	Overall	Adults	Pediatric	*p*-Value
	N = 193	N = 170	N = 23	
Age	41.8 ± 19.2	45.7 ± 16.9	12.9 ± 5.03	0.001
Sex (female)	133 (68.9%)	116 (68.2%)	17 (73.9%)	0.755
Follow-up period (months)	19.1 ± 33.2	19.4 ± 33.3	16.8 ± 33.5	0.21
Angioedema	101 (52.3%)	93 (54.7%)	8 (34.8%)	0.116
Atopic background	41 (21.2%)	34 (20.0%)	7 (30.4%)	0.381
Atopic dermatitis	13 (6.7%)	9 (5.3%)	4 (17.4%)	0.0838
Asthma	20 (10.4%)	15 (8.8%)	5 (21.7%)	0.123
Rhinitis	18 (9.3%)	15 (8.8%)	3 (13.0%)	0.786
Conjunctivitis	4 (2.1%)	4 (2.4%)	0 (0%)	1
Total co-morbidities	85 (44.0%)	83 (48.8%)	2 (8.7%)	0.001
Metabolic syndrome	61 (31.6%)	61 (35.9%)	0 (0%)	0.00122
Psychosomatic/neurological	27 (14.0%)	25 (14.7%)	2 (8.7%)	0.646
Malignancy	20 (10.4%)	19 (11.2%)	1 (4.3%)	0.52
Autoinflammatory	3 (1.6%)	3 (1.8%)	0 (0%)	1
Dermatological (psoriasis)	7 (3.6%)	7 (4.1%)	0 (0%)	0.691
Familial Mediterranean Fever family history	5 (2.6%)	5 (2.9%)	0 (0%)	0.908
Urticaria family history	11 (5.7%)	10 (5.9%)	1 (4.3%)	1
Atopy family history	34 (17.6%)	30 (17.6%)	4 (17.4%)	1
Family autoimmunity	6 (3.1%)	4 (2.4%)	2 (8.7%)	0.3
Aspirin use	8 (4.1%)	8 (4.7%)	0 (0%)	0.613
Disease triggers	26 (13.5%)	19 (11.2%)	7 (30.4%)	0.0269
Chronic inducible urticaria (physical component)	23 (11.9%)	16 (9.4%)	7 (30.4%)	0.00994
Chronic inducible urticaria (hormonal component)	3 (1.6%)	3 (1.8%)	0 (0%)	1

N = number of patients.

**Table 2:** Autoimmune co-morbidities.

Parameter	Overall	Adults	Pediatric	*p*-Value
	N = 193	N = 170	N = 23	
Total autoimmune co-morbidities	67 (34.7%)	59 (34.7%)	8 (34.8%)	1
Autoimmune diseases	28 (14.5%)	22 (12.9%)	6 (26.1%)	0.172
Hashimoto’s disease	4 (2.1%)	3 (1.8%)	1 (4.3%)	0.971
Hypothyroidism	15 (7.8%)	14 (8.2%)	1 (4.3%)	0.811
Bechet’s disease	1 (0.5%)	1 (0.6%)	0 (0%)	1
Graves’ disease	2 (1.0%)	2 (1.2%)	0 (0%)	1
Autoimmune liver disease	2 (1.0%)	0 (0%)	2 (8.7%)	0.00564
Vitiligo	1 (0.5%)	1 (0.6%)	0 (0%)	1
Systemic lupus erythematosus	1 (0.5%)	1 (0.6%)	0 (0%)	1
Sjogren’s syndrome	2 (1.0%)	2 (1.2%)	0 (0%)	1
Mixed connective tissue disease	1 (0.5%)	1 (0.6%)	0 (0%)	1
Scleroderma	1 (0.5%)	1 (0.6%)	0 (0%)	1
Inflammatory bowel disease	5 (2.6%)	2 (1.2%)	3 (13.0%)	0.00774
Antiphospholipid antibodies	4 (2.1%)	4 (2.4%)	0 (0%)	1
Bullous pemphigoid	1 (0.5%)	1 (0.6%)	0 (0%)	1
Autoantibodies	55 (28.5%)	50 (29.4%)	5 (21.7%)	0.786
Anti-nuclear antibody	37 (19.2%)	34 (20.0%)	3 (13.0%)	1
Anti-double-stranded DNA	7 (3.6%)	7 (4.1%)	0 (0%)	1
Perinuclear ANCA	3 (1.6%)	2 (1.2%)	1 (4.3%)	0.15
Anti-thyroid peroxidase	22 (11.4%)	20 (11.8%)	2 (8.7%)	1

N = number of patients.

**Table 3:** Laboratory tests results.

Parameter	Overall	Adults	Pediatric	*p*-Value
	N = 193	N = 170	N = 23	
Elevated platelets (>440 K/µL)	5 (2.6%)	3 (1.8%)	2 (8.7%)	0.0096
Hemoglobin (females 11.7–15.7 g/dL, males 13.5–17.5 g/dL)	127 (65.8%)	116 (68.2%)	11 (47.8%)	0.452
Elevated white blood cells (>10.8 K/µL)	46 (23.8%)	45 (26.5%)	1 (4.3%)	0.213
Elevated eosinophils (>0.5 K/µL)	7 (3.6%)	5 (2.9%)	2 (8.7%)	0.174
Elevated TSH (>4 mIU/L)	10 (5.2%)	8 (4.7%)	2 (8.7%)	0.05
Low TSH (<0.4 mIU/L)	14 (7.3%)	14 (8.2%)	0 (0%)	
Elevated C-reactive protein (>5 mg/L)	85 (44%)	81 (47.6%)	4 (17.4%)	0.575
Elevated immunoglobulin E (>188 IU/mL)	37 (19.2%)	32 (18.8%)	5 (21.7%)	0.113
Stool parasites	7 (3.6%)	7 (4.1%)	0 (0%)	0.871

N = number of patients.

**Table 4:** Treatment protocol.

Parameter	Overall	Adults	Pediatric	*p*-Value
	N = 193	N = 170	N = 23	
First-generation antihistamines	85 (44.0%)	83 (48.8%)	2 (8.7%)	0.001
Second-generation antihistamines	175 (90.7%)	154 (90.6%)	21 (91.3%)	1
Antihistamines Standard dose × 1	27 (14.0%)	20 (11.8%)	7 (30.4%)	0.00591
Antihistamines Standard dose × 2	74 (38.3%)	66 (38.8%)	8 (34.8%)	
Antihistamines Standard dose × 3	27 (14.0%)	27 (15.9%)	0 (0%)	
Antihistamines Standard dose × 4	63 (32.6%)	56 (32.9%)	7 (30.4%)	
Oral steroids	128 (66.3%)	119 (70.0%)	9 (39.1%)	0.007
Intravenous steroids	60 (31.1%)	60 (35.3%)	0 (0%)	0.001
Cyclosporine	9 (4.7%)	9 (5.3%)	0 (0%)	0.546
Omalizumab	46 (23.8%)	42 (24.7%)	4 (17.4%)	0.609
Other treatments (Montelukast, Colchicine, Dapsone, Methotrexate, and Azathioprine)	102 (52.8%)	94 (55.3%)	8 (34.8%)	0.104

N = number of patients.

## Data Availability

Data is unavailable due to privacy and ethical restrictions. Any data requested will be available via the corresponding author.
